# Identification of People with Diabetes Treatment through Lipids Profile Using Machine Learning Algorithms

**DOI:** 10.3390/healthcare9040422

**Published:** 2021-04-06

**Authors:** Vanessa Alcalá-Rmz, Carlos E. Galván-Tejada, Alejandra García-Hernández, Adan Valladares-Salgado, Miguel Cruz, Jorge I. Galván-Tejada, Jose M. Celaya-Padilla, Huizilopoztli Luna-Garcia, Hamurabi Gamboa-Rosales

**Affiliations:** 1 Unidad Académica de Ingeniería Eléctrica, Universidad Autónoma de Zacatecas, Jardín Juárez 147, Centro, Zacatecas 98000, Mexico; vdrar.06@uaz.edu.mx (V.A.-R.); alegarcia@uaz.edu.mx (A.G.-H.); gatejo@uaz.edu.mx (J.I.G.-T.); jose.celaya@uaz.edu.mx (J.M.C.-P.); hlugar@uaz.edu.mx (H.L.-G.); hamurabigr@uaz.edu.mx (H.G.-R.); 2 Unidad de Investigación Médica en Bioquímica, Hospital de Especialidades, Centro Médico Nacional Siglo XXI, Instituto Mexicano del Seguro Social, Av. Cuauhtémoc 330, Col. Doctores, Del. Cuauhtémoc, Mexico City 06720, Mexico; adan.valladares@imss.gob.mx (A.V.-S.); miguel.cruzlo@imss.gob.mx (M.C.)

**Keywords:** type 2 diabetes, diabetic treatment, logistic regression, random forest, K-nearest neighbor, decision trees, computer-aided diagnosis, statistical analysis

## Abstract

Diabetes incidence has been a problem, because according with the World Health Organization and the International Diabetes Federation, the number of people with this disease is increasing very fast all over the world. Diabetic treatment is important to prevent the development of several complications, also lipid profile monitoring is important. For that reason the aim of this work is the implementation of machine learning algorithms that are able to classify cases, that corresponds to patients diagnosed with diabetes that have diabetes treatment, and controls that refers to subjects who do not have diabetes treatment but some of them have diabetes, bases on lipids profile levels. Logistic regression, K-nearest neighbor, decision trees and random forest were implemented, all of them were evaluated with accuracy, sensitivity, specificity and AUC-ROC curve metrics. Artificial neural network obtain an acurracy of 0.685 and an AUC value of 0.750, logistic regression achieve an accuracy of 0.729 and an AUC value of 0.795, K-nearest neighbor gets an accuracy of 0.669 and an AUC value of 0.709, on the other hand, decision tree reached an accuracy pg 0.691 and a AUC value of 0.683, finally random forest achieve an accuracy of 0.704 and an AUC curve of 0.776. The performance of all models was statistically significant, but the best performance model for this problem corresponds to logistic regression.

## 1. Introduction

Diabetes is part of a diseases set called Noncommunicable Diseases (NCDs), it is known as a chronic disease, two important characteristics are that it is of a long duration and it is the result of a scheme of several factors as: genetic, environmental and behaviours factors [1]. Diabetes is characterized by hyperglycemia, it refers to a complex disorder that involve profound alterations in the metabolism of fats, proteins and carbohydrates, resulting from defects in insulin secretion, insulin action, and even both of them [2,3]. There are several problems that can be developed like: long-term damage, failure or dysfunction of various organs, vascular complications, all of them shorten the life expectancy of who is diagnosed with this disease [4].

The control of diabetes and the problems that can be developed is the main objective for doctors and diagnosed patients, for that reason, the patient must have a diabetes treatment. Antidiabetic drugs are pharmacological agents that have been approved for hyperglycemic treatment in diabetes type 2. These drugs are classified as biguanides, sulfonylureas, meglitinides, thiazolidinediones, dipeptidyl peptidase IV inhibitors and α-glucosidase inhibitors [5].

The lipid control levels are important in diabetic patients [6]. A high cholesterol level can lead to the accumulation of plaques on the walls of blood vessels, and this can block arteries and cause high blood pressure, stroke, heart disease, or heart attack. High triglycerides are associated with the risk of developing metabolic syndrome, which can increases the risk of heart disease and other disorders, including diabetes [7,8].

Recent studies in the health area, have been adopting machine learning and deep learning algorithms, due to the high performance in several healthcare applications, this is part of diseases diagnosis or classification making implementations of algorithms based on computer-aided diagnosis (CAD) where prediction models are used when it is necessary to know in the future the behavior of some data related to any disease, for example diabetes [9]. Machine learning techniques are implemented to discover patterns from medical data sources and provide excellent capabilities to predict diseases or classify diseases [10].

On the other hand, it is important to mention that diabetes incidence has been a problem, because according with the World Health Organization (WHO) and the International Diabetes Federation (IDF), the number of people with this disease is increasing very fast all over the world [1]. Despite the impact that diabetes has had on society and the efforts made to design effective therapeutic protocols and drugs, it has been documented that most current therapies for this disease are developed in the absence of defined molecular targets or a complete delineation of the pathogenesis of the disease. For this reason and due to the continuous increase in knowledge of pathophysiological mechanisms and the side effects of therapeutic protocols, drug design and discovery has become a major challenge in the field of diabetes research. The contribution of data mining and machine learning in this area are focused on helping in different aspects, such as: making recommendations and improvements to the effectiveness of medication, making predictions, as well as suggestions for more personalized medications, also helping to design more effective blood glucose reduction factors, as well as improving the planning and dosage of medications, and applying the administration of drugs in a more specific way [11].

For that reason, the main objective of this study is to analyze the relation that exists between the diabetes treatment and lipids profile, implementing machine learning algorithms.

The contribution of this work is that given the lipids profile, age and gender, the machine learning algorithm can determine if a person is on diabetes treatment, because there were five machine learning algorithms implemented, and based on a comparative, it can determine which of them provided the best model to give a solution to this problem, permitting us to know if there is a relationship between subjects who have or do not have diabetic treatment and their lipids profile, being a first approach to help with the lipids profile control in subjects with type 2 diabetes and how the medication modifies parameters to optimize the treatment developing a computational assisted diagnosis (CAD). Due to it being considered an important first step for future research in this area.

### Related Work

Diabetes represents one of the greatest challenges of this century, because it is a major cause of death and disability worldwide [12]. Mainly type 2 diabetes, due to it being an expanding health problem [13]. This disease is influenced by genetic risk and diverse environmental factors [14].

For that reason, there are some machine learning approaches focused in this disease. A related work uses machine learning paradigm to detect diabetes disease, National Health and Nutrition Examination Survey (NHANES 2009–2012) diabetes dataset was used, they implemented naïve bayes, decision tree, adaboost and random forest to predict diabetes disease. The highest accuracy results were obtained from a combination of logistic regression and random forest, that was 94.15% and an AUC of 0.94 [15].

Another approach consist in the classification of diabetic patients through lipids profile levels, Guerrero-Flores et al. [16] implemented three algorithms, which are logistic regression, decision trees and support vector machine, the AUC values obtained are from 0.613 to 0.727.

Almatrooshi et al. [17] proposed an integration of two systems to create a system that is able to detect diabetes and after that recommend a proper plan or medication to overcome diabetes, they evaluated and tested four different approaches to detection diabetes, the most accurate approach was random forest with an accuracy of 79.2% and F-measure of 0.787.

On the other hand, Koren et al. [18] decided to test the utility of machine learning applied to big data, specifically in the identification of the potential role of concomitant drugs not taken for diabetes which may contribute to lowering blood glucose. They implemented logistic regression to predict the probability of treatment success with the matched drug and this constituted the propensity score. The basis metric was HgA1c, then patients with levels <6.5 were classified as successfully treated and according to the results, 54% of the patients were successfully treated.

In the approach proposed by Alcala-Rmz et al. [19] 19 para-clinical features were used to determine the health status of the patients. Among the 19 features there were the lipids profile of each subject. They developed a model that was evaluated through a statistical analysis based on the calculation of the loss function, accuracy, area under the curve (AUC) and receiving operating characteristics (ROC) curve. This model was able to obtain an accuracy of 0.94, and AUC values of 0.98.

Hosseini et al. [20] proposed an algorithm that is based on the notion of Markov blankets in Bayessian networks. They applied the algorithm with the aim to optimize medication prescriptions for diabetic patients, taking in count different features, for example if the patient suffers of multiple comorbidities and if the subject is currently taking multiple medications. With this study, they evaluated the features with a bayessian network, and achieve a precision of 88.75% and an AUC of 71.15%

The objetive of the Wu et al.’s [21] study, is to assess several machine learning algorithms and screen out a model that can be used to predict patients’ non-adherence risks. For this work, 401 patients were selected from 630 candidates, of which 85 were evaluated as poor adherence (21.20%). A total of 16 featured were evaluated in the model, 300 models were built based on 30 machine learning algorithms. The highest results corresponds to an AUC of 0.866.

Kowsher et al.’s [22] research consist of a comparative study of 7 machine learning classifier algorithms and an artificial neural network approach to predict the detection and treatment of diabetes. The traininig dataset was comprised by the information of 9483 diabetic patients. The performance evaluation metrics were accuracy and precision, looking for the best algorithm, the best accuracy performance was from artificial neural network with 95.14%.

In the Wright et al.’s study, the sequential pattern mining approach is used, the main objective was to identify the temporal relationships between medication prescriptions, and in this way predict the follow-up medication to be prescribed for a patient [23].

Finally, Oh et al. proposed a novel method to follow trajectories, the method was focused on studying the trajectories of type 2 diabetes, using electronics health record systems. They were able to identify the trajectory that most people follow, which consists of a sequence of diseases ranging from hyperglycemia to hypertension, impaired fasting glucose and type 2 diabetes [24].

In recent years there are many related works that involves machine learning as a useful tool for many approaches that pretend to give a solution to the diabetes problem, as mentioned before, machine learning is being used with different objectives from the classification of diabetes patients to determining the optimal diabetic treatment for diabetic patients, all of them pretend to give solutions to society as far as this disease is concern. In addition, some related work has found hyperlipidemia to be a relevant factor in the study of type 2 diabetes. In this paper we propose the implementation of five machine learning classifiers, which are neural networks, logistic regression, k-nearest neighbor, decision trees and random forest that are able to classify, if a subject has a diabetic treatment or if a subject does not have treatment through lipids profile values like: total cholesterol, High Density Lipoprotein (HDL), Low Density Lipoprotein (LDL) and triglycerides.

## 2. Materials and Methods

The methodology followed to analyze the database provided by the Medical Research Unit in Biochemistry, “Centro Médico Siglo XXI”, “Instituto Mexicano del Seguro Social”, is presented in this section, as well as the data description, data preprocessing, data classification and the validation of the results. The study focuses on the classification of subjects who have diabetes treatment and subjects who do not have the treatment.

**Figure 1:**
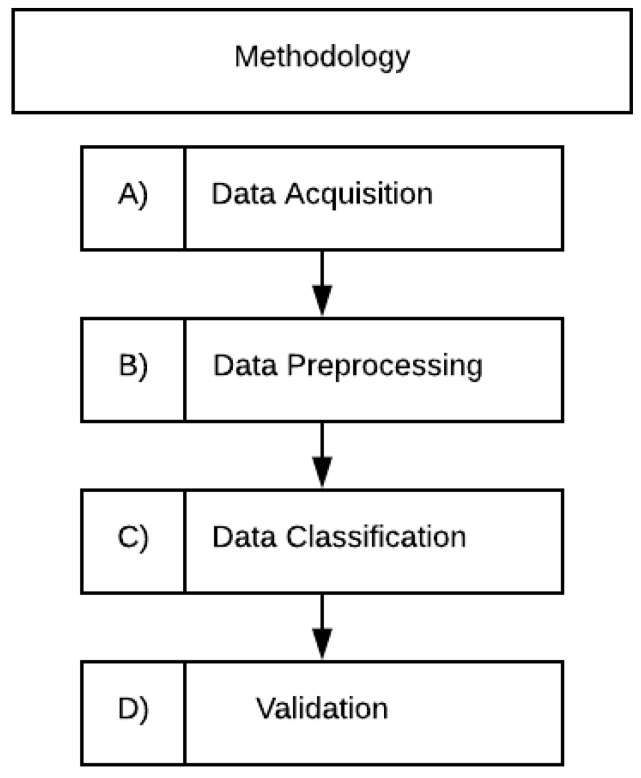
Flowchart of the methodology followed.

The methodology flowchart is shown in Figure 1. Section (A) corresponds to data acquisition. The next section (B) refers to data preprocessing, in this stage some techniques were implemented to analyze the database, solve outliers problems and separate the data in two subsets. In section (C) the implemented machine learning algorithms is explained: neural networks, logistic regression, K-nearest neighbor, decision tree and random forest. Finally, section (D) represents the validation process for each model, using statistical parameter like sensitivity, specificity, AUC, ROC curve and accuracy.

### 2.1. Dataset Description

**Figure 2:**
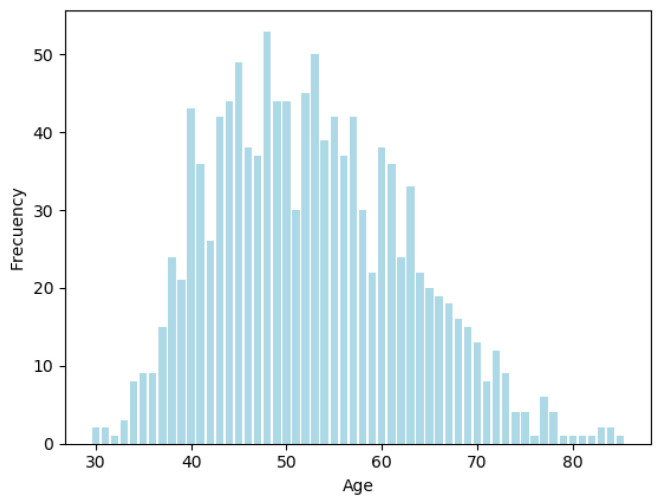
Age distribution.

The dataset contains information referring to 1198 Mexican subjects, 537 cases that have diabetes and diabetes treatment, and 661 controls who do not have diabetes treatment but some of them have diabetes and some of them do not have diabetes. Also, in the dataset there are 499 males and 561 females, Figure 2 corresponds to age distribution, the age range of the dataset is between 30 and 85 years old.

Also the dataset is composed by another 4 input features, which are described in Table 1. The output feature indicates “1” whether a subject has diabetes treatment or “0” does not have treatment. The cases treatment includes metformin, glibenclamide, pioglitazone, roziglitazone, acarbose or insuline.

### 2.2. Data Preprocessing

The dataset features were normalized using z-score method, this method consists of transforming the data to a distribution with mean 0 and standard deviation 1. Z-score method was used with two purposes, the first one was to define the same numeric scale for the data, the second one was to identify outliers in the dataset, because while calculating the Z-score the data were re-scaled and centered and there were looking for data points which were too far from zero, then the data points which were way too far from zero were treated as the outliers. A threshold of 3 or −3 was used, because it is the commonly used [25]. Also the boxplots was used to visualize the data distribution before and after to removing outliers for each feature.

Boxplot is known as a “box and whisker plot” and it is a method to identify outliers. The diagram is comprised by a box with the interquartile range (IQR), the box has a line horizontally in the middle, this represents the median score [25,26]. On the other hand, there is an upper quartile which represents the 75th percentile, also the bottom of the box shows the lower quartile which represents the 25th percentile. Finally, the long extensions from the box represents the highest and lowest values in the expected normal distribution [27,28].

**Figure 3:**
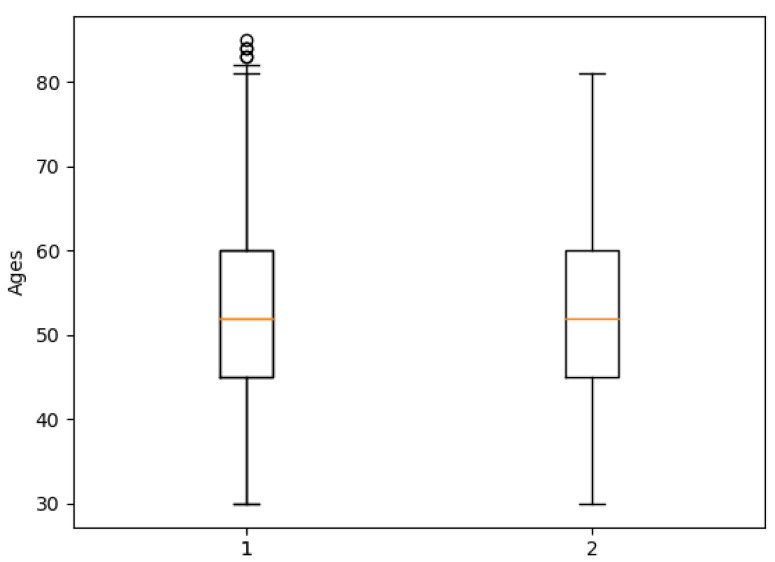
Age Boxplots.

Figure 3 shows the boxplot for subjects ages, the number 1 represent the boxplot ages before deleting the outliers and number 2 refers to boxplot ages after removing outliers. People over 80 years old were deleted from the dataset.

**Figure 4:**
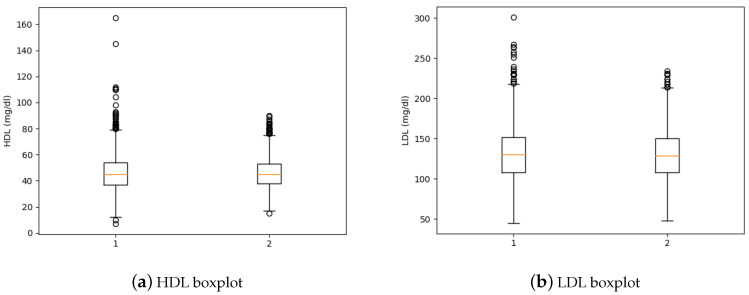
HDL and LDL Boxplots.

Figure 4a presents the boxplot for HDL and Figure 4b shows the boxplot for LDL features, in both cases 1 corresponds to boxplot before removing the outliers and number 2 represents the boxplot after deleting outliers.

**Figure 5:**
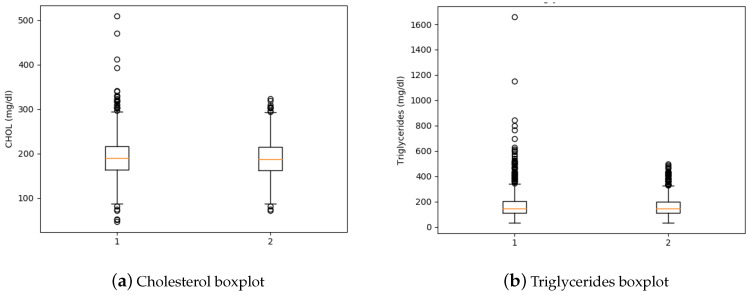
Cholesterol and Triglycerides Boxplots.

Finally, the boxplots for total cholesterol are shown in Figure 5a and the boxplots for triglycerides are presented in Figure 5b. As in the previous diagrams, the number 1 indicates the boxplot before deleting the outliers and number 2 refers to boxplot after removing outliers, according to the threshold selected.

Once the outliers corresponding to the chosen threshold have been eliminated, the dataset decreased from 1198 subjects to 1060, where 459 patients have diabetes and diabetes treatment and 601 subjects who do not have diabetic treatment but some of them have diabetes.

Due to the dataset size, it was decided to perform a blind test, which consists of dividing the data set into two subsets, one for training and the other for testing. Then, the main dataset was randomly divided in two sets. The first one refers to the training set, involving 70% of all dataset, and the second was the test set, this subset corresponds to the remaining 30%.

#### 2.2.1. Data Classification

In this section it is explained the machine learning algorithms, the tools and packages used to the classification stage.

The subjects who do not have a diabetes treatment are labeled as “0”, which are control subjects, the case subjects were labeled with “1” and corresponds to patients with diabetes treatment. The implemented algorithms were: neural networks, logistic regression, K-nearest neighbor, decision tree and random forest, using the scikit-Learn, keras and tensorflow packages, for Python.

- Scikit-Learn: is a Python library that provides different supervised and unsupervised learning algorithms to solve regression, classification, clustering problems, etc. It is built upon packages like Numpy, Pandas, Scipy and matplotlib [29].
- Keras: is known as a high-level Artificial Neural Network API, which is written in Python, designed specifically to enable fast experimentation with deep neural networks, it focuses on being user-friendly, modular, and extensible [30].
- Tensorflow: is an open-source software library for dataflow programming. It is a symbolic math library. Furthermore it is used for machine learning applications such as neural networks [31].
- Deep Neural Network: Neural Networks looking for a solution using a correlation between features. Neural Networks acquire their knowledge by detecting relationships and patterns between the data, learning through experience. It is composed by hundreds of neurons that can be modifiable, connected with weights, organized in layers, which can also be modified. Learning rule, transfer functions and architecture itself are parameters that determine the deep neural network behavior [32]. Two activation functions were used, the first one was Rectified Linear Unit (ReLU), which is shown in Equation (1), it was implemented in all dense layers, except outter layer, the function assigns “0” to neurons that have a value lower than “0”, and the original value is assigned when the value is above or equal to “0” [33].
  (1) $$ReLu(z)=\left\{\begin{array}{cc} \mathrm{if}z<0 & 0\\ \mathrm{if}z\geq 0 & z \end{array}\right.$$
  The second function was softmax based on general logistic function, represented in Equation (2) where σ(z) is a *K*-dimensional vector of *z*. It gives a vector of arbitrary values located between 0 and 1 [34].
  (2) $$\sigma {\left(z\right)}_{j}=\frac{e^{z_{j}}}{\sum_{K=1}^{K}e^{z_{K}}},j=1,...,K$$
  In this study, the independent variables are; age, gender, hdl, ldl, chol and tg, the dependent variable is tx, that indicates if the subject has or does not have diabetes treatment. The main parameters used were 5 dense layers, with 100 neurons each one, except the last one that corresponds to the output and has 2 neurons; 3 dropout layers, with a rate of 0.25, 0.50 and 0.25 respectively; the optimizer implemented was “Adam” and 100 epoch.
- Logistic Regression: consists of measuring the relationship between the categorical dependent variable and the independent variables, by estimating probabilities using a logistic function. It is used to predict binary response based on one or more predictor variables. In general terms, logistic regression can be defined as is shown in Equation (3), where *p* is the chance of the distinctive of interest [35,36].
  (3) $$\mathrm{logit}\left(p\right)=b_{0}+b_{1}X_{1}+b_{2}X_{2}+b_{3}X_{3}+...+b_{k}X_{k}$$
  The model obtained by the logistic regression permit us to know the relationship between the output, which corresponds to “0” if the subject is not taking antidiabetic medication or “1” does not have treatment; and the entry values, which are age, gender, hdl, ldl, chol an tg; through the analysis of the results, it is possible to know if the relationship exits or not.
- K-Nearest Neighbor (K-NN): is a simple classification algorithm, based on a distance metric, that evaluate the similarity of two features vectors. The main objective is to select the class label for the new input, which appears frequently in its *k* near neighbors. In other words, the purpose is computing similarities between unknown sample and training samples, the idea is to find the top *k* nearest neighbors of the unknown sample [37]. The K-NN algorithm has four principal steps [38,39]:
  - Select a “K” value (Neighbors)
  - For each example, calculate the distance between the query example and the current example from the data. After that, add the distance and the index of the example to an ordered collection. This distance is called Euclidean distance, which is shown in Equation (4), where *D*(*a*,*b*) means Euclidean length between *b* and *a* [40].
  - Sort the calculated distances in ascending order.
  - Obtain the top *k* rows from the sorted array.
  - Get the most frequent class.
  - Return the predicted class.
  (4) $$D\left(a,b\right)=\sqrt{\sum_{i=1}^{n}{\left(b\_i-a\_i\right)}^{2}}$$
  In this study the K-NN algorithm is implemented, k_neighbor = 16, the same 6 input features are used and in the same way, the output feature is “tx”, which indicates if a subject has diabetes treatment or not.
- Decision Tree: refers to a machine learning model, commonly used in classification problems. The algorithm analyzes the data by making decisions based on asking a series of question. It is a classifier model that correspond to a supervised learning algorithm [41,42]. In other words, this is a predictive model, which is used effectively to classify datasets. This model determines the best decisions in the analysis process, splitting the data into subset. In the learning stage, the model manages to maximize the information gain *I* in a given node, and it is represented as in Equation (5).
  (5) $$I=H\left(S\right)-\sum i\epsilon L,R\frac{|S{}^{i}|}{\left|S\right|}H\left(S^{i}\right)$$
- Random Forest (RF): it is an algorithm widely used in medical areas, is a supervised method that uses multiples decision trees to create a forest. RF builds multiple decision trees and merge them into a single tree, the purpose is to achieve a high prediction accuracy. In this model, there are settings that constructed many classification and regression trees using randomly selected training datasets and random subsets of prediction variables for modeling outcomes, then the results from the tree are added to give a prediction [43]. It is possible to use entropy to determine how nodes branch in a decision tree, as is shown in Equation (6), taking into consideration the probability of a certain outcome in order to make a decision on how the node should branch.
  (6) $$\mathrm{Entropy}=\sum_{i=1}^{C}-p_{i}*\log_{2}\left(p_{i}\right)$$

The algorithms were implemented in Python, which is an interpreter, object-oriented, high-level programming language with dynamic semantics. It can be used for a variety of applications, one of them is the data analysis [44].

#### 2.2.2. Evaluation

In the evaluation stage the metrics used to compare the models performance are presented. The outputs are presented in a confusion matrix, where the diagonal represents the observations that are correctly classified and the values outside of diagonal corresponds the observations that were incorrectly classified, also the class error for each model is calculated, based on confusion matrix values. In addition the accuracy and the Receiver Operating Characteristic (ROC) were calculated for each machine learning algorithm implemented.

The accuracy metric calculates the average performance of the algorithms, the purpose of this metric is to calculate the percentage of samples that are correctly classified as is shown in the Equation (7), where *TP* corresponds to true positives, *TN*, true negatives, *FP* false positives and *FN* false negatives.

(7) $$\mathrm{Accuracy}=\frac{\mathrm{TP}+\mathrm{TN}}{\mathrm{TP}+\mathrm{TN}+\mathrm{FP}+\mathrm{FN}}$$

In addition, the ROC curve is a parameter used to measure the classification precision of the model, trough the sensitivity and specificity. Sensitivity refers to the proportion of subjects with a positive condition that were correctly classified and it is calculated with Equation (8), where *TP* are the true positives and *FP* are the false positives [45].

(8) $$\mathrm{Sensitivity}=\frac{TP}{TP+FN^{\prime }}$$

Specificity refers to the proportion of true negatives, which means the subjects with a negative condition that were correctly classified and it is calculated with Equation (9), where *TN* are the true negatives and *FN* are the false negatives [45].

(9) $$\mathrm{Specificity}=\frac{TN}{FN+TP^{\prime }}$$

The ROC curve is used to visualize the performance of the classifiers, this is complemented with the area under the ROC curve (AUC), this complement represents the probability that a random positive sample is correctly identified.

In this study the ROC curves were calculated for each classifier algorithm.

The implementation of this work was performed with a laptop DELL g7, Intel Core i7-8750H 2.20 GHz, 16 GB, 500 GB SSD, Windows 10, 64-bit; and with the version of Python 2.7.

## 3. Results

In this study a dataset is used with a total of 7 features, which 6 of them are the input data for the classifiers, and the remaining feature is the output feature, which indicates with the label “0” the absence of diabetic treatment and with label “1” if the patient has a diabetic treatment. The main objective is to look for the machine learning algorithm with the highest performance in the binary classification explained above.

In the preprocessing step, they were analyzed with boxplot and *z*-score, the possible outliers for each feature, a threshold was also selected to remove the points data which were too far from zero, for this reason the dataset was reduced from 1198 subjects to 1060, where 459 patients were cases and 601 subjects were control. After this step, the dataset was divided in two subsets, one for training, containing 70% of the data (421 controls/321 cases), and one for testing containing 30% of the data (180 controls/138 cases).

Once finished the preprocessing stage, the implementation of each classifier was carried out, it was calculated the accuracy, sensitivity, specificity and AUC value for each model. In Table 2 the classifier name and the values obtained in each metric are presented. Logistic regression achieved the best performance according to the metrics values obtained, followed by random forest, which got an accuracy value of 0.704 and an AUC value of 0.776, this means that the model is able to classify 77.6% of the data correctly, after that, neural network obtained an accuracy of 0.685 and an AUC of 0.750. The K-NN and Decision Tree obtained the worst performance, but both models got a statistical significance values.

**Figure 6:**
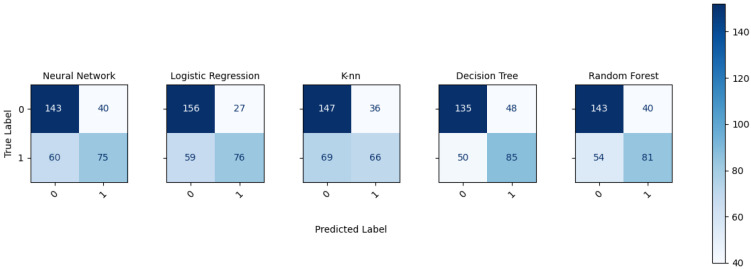
Confusion matrix of each implemented algorithm.

In addition, the confusion matrix of each implemented algorithm are presented in Figure 6. The diagonal of each matrix contains the predictions that were correctly classified, and the off-diagonal of each matrix represents the observations that were incorrectly classified.

Furthermore, Table 3 shows the error class for each algorithm, the error was calculated from the confusion matrix values, the minimum error value is presented by logistic regression for class 0 and the minimum error value is presented by decision tree for class 1.

**Figure 7:**
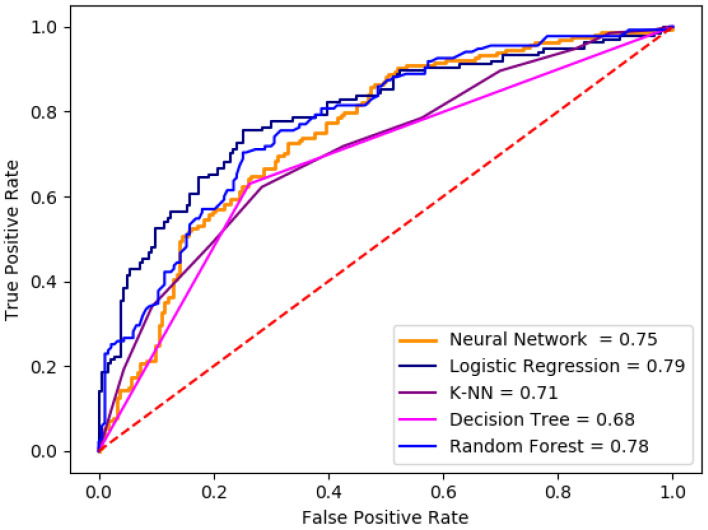
ROC curves obtained with the average performance of each implemented model.

Also, each model was validated calculating their ROC curves based on the performance of each model, which are presented in Figure 7. The ROC curve for neural network is presented in orange line, with an AUC value of 0.75. ROC curve for the logistic regression is presented in dark blue, with an AUC value of 0.79. The ROC curve for k-nn model is shown in blue color, with an AUC value of 0.71. The ROC curve calculated for decision tree classifier is presented in pink line, obtaining an AUC value of 0.68, and finally, the ROC curve calculated for random forest model is presented in purple, with an AUC value of 0.78.

The performance of logistic regression and random forest models was similar, on the other hand, the performance of k-nn and decision tree were lower, but with a statistical significance in this area.

## 4. Discussion

The related work indicates the importance of analyzing the drugs that are prescribed to diabetic patients, there are different approaches, but all of them approach the same objective, which is to know the impact that drugs have on patients, as well as to find patterns that help the pharmaceutical industry to improve drugs, based on the needs that arise. This work is a first approach to know the impact that different diabetic medications have on patient’s health, specifically within the lipid profile, because an uncontrolled lipid level can lead to different complications [7,8].

In this work five machine learning algorithms are implemented: neural networks, logistic regression, K-NN, decision tree and random forest; each model was validated through accuracy, sensitivity, specificity, AUC and ROC curves metrics.

The evaluated dataset is comprised by 1060 Mexican subjects, where 459 are subjects diagnosed with diabetes that follow a diabetic treatment and the remaining 601 are control subjects with or without diabetes but none of them follow a diabetic treatment. The features analyzed are: Age, Gender, HDL, LDL, Cholesterol, Triglycerides and Treatment (TX), it is important to mention that these features were selected with the aim to classify subjects with diabetes treatment from subjects who do not have diabetic treatment, through their lipids profile.

The highest accuracy obtained in the testing stage corresponds to logistic regression model, which achieve an accuracy of 0.729 with a sensitivity of 0.852 and 0.795 in AUC metric, there is no big difference between this model and random forest model, due to its performance it indicates an accuracy of 0.704, a sensitivity value of 0.781 and 0.776 in the AUC metric, another model with high performance is neural network. which performance corresponds to an accuracy of 0.685, 0.781 of sensitivity and an AUC of 0.750. These three models are able to classify more than 70% of subjects correctly. K-NN and decision tree are not far from the other two, both of them achieve significant values that are over 66% in accuracy, sensitivity and AUC metrics.

In addition, Figure 7 shows that all curves presented statistically significant values > 67%. These curves refers to the proportion of true positives and true negatives.

On the other hand, this study demonstrate that it is possible to identify subjects who have diabetes treatment from those who do not have diabetes treatment, showing the relevance of cholesterol, HDL; LDL and triglycerides features. The models obtained can be useful for doctors to know if patients are following the established treatments correctly or simply know if a new patient has been taking an antidiabetic drug, allowing for a better control of the patient’s medical history. Furthermore, according to [46] subjects that are taking sulphonylurea therapy, have observed effects on their lipids profile, also a group treated with insulin along with metformin had significant improvement in the lipids levels. Because, once that the Hemoglobin A1c (HbA1c) is improved through diabetic treatment, the lipids profile can significantly improve [47].

It is important to remember that the dataset is composed by mexican subjects information, for this reason, the results can be implemented in tools that allow to improve the Mexican health.

Another advantage of the models implemented is that they do not require high computational cost, because it is not necessary to acquire a special equipment.

## 5. Conclusions

The results obtained in this work, permits us to conclude that the database is adequate for the aim in this study, also, it allows to classify subjects who have a diabetic treatment or that do not have a diabetic treatment, based on the lipids profile, age and gender.

On the other hand, specificity achieves a low value for each model, it is necessary to remember that in the medical area it is more important to find true positives states, because the sensitivity metric should be higher.

Also, it is possible to develop a tool based on lipids profile to detect whether a subject has a diabetes treatment or not, implementing any of the models obtained in this study.

The ROC curves in Figure 7 shows that decision tree is one of the worst AUC with 0.68, which means that only 68% of the subjects were correctly classified, but it is important to mention that the performance values might be improved increasing the observation numbers in the dataset. Besides, logistic regression was able to classify 79% of the total subjects, random forest has the second place, because it achieved a 78% of the subjects correctly classify, neural network was able to classify 75% of the subjects in the correct way and K-NN obtained an AUC value of 0.71, which means that 71% of the subjects in the dataset were classified correctly.

In addition, the results obtained demonstrate that the lipids profile is an important feature in this classification, because it can be modeled by the classifiers implemented, also the results show a relationship between lipids profile and a subject with diabetic treatment.

This work is considered an important basis to search for a specific relationship between the different medications prescribed to a diabetic patient and the impact they have on their lipid profile.

## 6. Future Work

As future work we propose to change the machine learning algorithms parameters, and also find a way to increase the database observation.

On the other hand, it could be interesting to implement other machine learning algorithms or apply a different classification approach, and it also could be important to do an analysis that shows in a clear way the relationship between lipids profile and the diabetic treatment of the subjects.

## Figures and Tables

**Table 1:** Description of features contained in the dataset.

Feature	Description
Age	Subject age
Gender	Subject Gender
CHOL	Total Cholesterol (mg/dL)
HDL	High Density Lipoprotein (mg/dL)
LDL	Low Density Lipoprotein (mg/dL)
TG	Triglycerides (mg/dL)
TX	0—absence of diabetic treatment 1—diabetic treatment

**Table 2:** Performance comparison based on accuracy and AUC.

Classifier	Accuracy	Sensitivity	Specificity	AUC
Neural Network	0.685	0.781	0.555	0.750
Logistic Regression	0.729	0.852	0.562	0.795
K-NN	0.669	0.803	0.488	0.709
Decision Tree	0.691	0.737	0.629	0.683
Random Forest	0.704	0.781	0.600	0.776

**Table 3:** Class error for each algorithm implemented, based on confusion matrix.

	Class Error	
	**0**	**1**
Neural Network	0.218	0.444
Logistic Regresion	0.147	0.437
K-nn	0.196	0.511
Decision Tree	0.262	0.370
Random Forest	0.218	0.400
